# Rubella Elimination and Improving Health Care for Women^1^

**DOI:** 10.3201/eid1011.040428

**Published:** 2004-11

**Authors:** Carlos Castillo-Solórzano, Jon Kim Andrus

**Affiliations:** * Pan American Health Organization Washington, DC, USA

**Keywords:** Elimination, Rubella, Benefits, Women, Health, Care, conference report

## Abstract

In the Americas, the Pan American Health Organization supports strategies for the appropriate control and elimination of vaccine-preventable diseases, especially if the strategies are designed to reduce health inequities, strengthen the political commitment for immunization services, and promote the culture of prevention. In September 2003, the 44th Directing Council of the Pan American Health Organization adopted a goal to eliminate rubella and congenital rubella syndrome by 2010. One of the main objectives of this initiative is improving women's health, consistent with achieving the Millennium Development Goals. An important component of rubella elimination is conducting vaccination campaigns for men and women of childbearing age to reduce rapidly the number of people susceptible to rubella infection. From 1998 to 2002, the English-speaking Caribbean, Chile, Costa Rica, Brazil, and Honduras conducted mass rubella vaccination campaigns aimed at adults. Vaccination coverage reached >95% in each country with an exception of the Caribbean, where the coverage was 80%.

## Abstract 2

Health care for women can be improved by strengthening adult health services, improving health awareness and community participation, decentralizing decision-making, and using epidemiologic surveillance.

The Pan American Health Organization (PAHO) supports strategies that encourage political commitment, reduce inequities in health, and enhance the culture of prevention (*1*). In that context, the initiative to eliminate rubella and congenital rubella syndrome was adopted by the directing council of PAHO in September 2003 (*2*). Political commitment to expand coverage and reach children and women who generally do not receive health services is important to the success of this initiative. The work of the national immunization program in the Americas is not limited to children and includes reaching the adult population, especially women of childbearing age.

The initial purpose of the Expanded Program on Immunization (EPI) in the Americas, launched in 1977, was to reduce illness and deaths from prevalent childhood diseases that could be prevented with vaccination (*3*). EPI accomplished this purpose by setting up and expanding permanent services within the framework of primary health care and by creating the necessary mechanisms for effective, large-scale application of existing knowledge and technology.

Planning and developing EPI led to eradicating polio and progress toward eliminating measles. The goal of eliminating neonatal tetanus as a public health problem was also met and sustained. Now, the region is challenged with eliminating rubella and congenital rubella syndrome by 2010. One of the main objectives is improving women's health, which is also one of the major Millennium Development Goals in health (*4*).

Eliminating polio from the Americas, with a reduction in the number of reported cases from 6,653 in 1970 to 0 in August 1991, is often sited as an example of effective collaboration among governments, nongovernmental organizations, the private sector, and local communities (*5*). Collaborators participated in implementing all aspects of operations, including financing, training, surveillance, vaccinating, and mobilizing mass media. The partners also helped mobilize volunteers to access hard-to-reach populations for vaccination.

The experience and concrete products obtained during the years of the polio elimination initiative benefited the ministers of health by giving them the necessary confidence and credibility for allocating adequate resources to the current vaccination programs and embarking on new initiatives. The countries now cover >80% of the expenditures of routine vaccination programs, including the purchase of vaccines.

Building on the successful experience of Cuba and English-speaking Caribbean countries in interrupting endemic transmission of the measles virus, the ministers of health adopted a resolution at the 24th Pan American Sanitary Conference in 1994 to interrupt indigenous measles transmission in the Americas by the year 2000. From 1990 to 2002, the number of reported measles cases declined by 99.2% in the Americas, with measles cases plummeting from ≈250,000 cases in 1990 to 2,109 cases in 2002 (*6*). Since September 2001, no viruses of the D6 strain have been identified in the Americas, and the last confirmed infection with d9 genotype was reported in November 2002. In 2003, 105 cases were reported. Of these, 44 were in Mexico and 45 in the United States (26 were indigenous and 19 imported).

As a consequence of administering at least two doses of tetanus toxoid to women of childbearing age in high-risk areas and ensuring that all cases were fully investigated, the number of reported cases of neonatal tetanus declined. In 2003, only 116 cases of neonatal tetanus were reported; ≈50% of them were in Haiti, the only remaining country in the Americas where neonatal tetanus continues to be endemic (*7*).

## Eliminating Rubella and Congenital Rubella Syndrome from the Americas by 2010

Cuba was the first country to eliminate rubella and congenital rubella syndrome. The last reported case of congenital rubella syndrome in Cuba occurred in 1989 and the last reported case of rubella in 1995. With measles elimination as a guide, similar strategies were proposed and implemented for rubella and congenital rubella syndrome elimination. This milestone was largely achieved by implementing two mass vaccination campaigns from 1985 to 1986. Initially, the Cubans tried to reach women 18–30 years of age and, then, children 1–14 years of age (*8*). In the rest of the region, the strengthening of measles surveillance demonstrated that circulation of rubella virus was widespread and that congenital rubella syndrome was an important public health problem (Figure). In response to the circulation of the rubella virus and the potential for the emergence of rubella epidemics, the countries of the Caribbean launched a subregional initiative in 1997 to eliminate rubella and congenital rubella syndrome.

**Figure:**
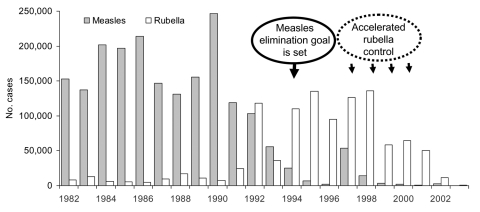
Number of confirmed measles and rubella cases, Americas, 1982–2003. Source: IM/Pan American Health Organization.

The adopted strategies included rapidly reducing the susceptible population and implementing high-quality surveillance. The specific strategies included using a rubella vaccine in routine childhood vaccination programs, aiming vaccination campaigns at men and women of childbearing age, developing integrated measles and rubella surveillance systems, implementing a congenital rubella syndrome surveillance system, and supporting improved laboratory capacity for isolating rubella virus (*9**,**10*).

Beginning in 1999, other countries accelerated their strategies for rubella control and the prevention of congenital rubella syndrome. Chile (1999), Costa Rica (2001), Brazil (2001–2002), Honduras (2002), and El Salvador and Ecuador (2004) have conducted mass rubella vaccinations among adults. They combined this strategy with the introduction of the rubella vaccine into their national childhood vaccination programs. This combination strategy is designed to rapidly reduce rubella virus circulation, while preventing a shift in the prevalence of the disease to susceptible young adults, especially women of childbearing age. The Caribbean, Costa Rica, Honduras, El Salvador, and Ecuador vaccinated men as well as women, while Chile and Brazil vaccinated only women of childbearing age. The countries that only vaccinated women have reported cases of rubella in 2004; the last congenital rubella syndrome case in Chile was in 2001 and in Brazil in 2004 (*11*).

By September 2004, a total of 43 countries and territories in the region had introduced vaccines containing the rubella antigen (MR or MMR) in their national child vaccination program. The only remaining country, Haiti, should do so in 2005.

## Rubella and Congenital Rubella Syndrome Elimination

The experiences of Cuba and the English-speaking Caribbean countries strengthened the commitment for rubella elimination in Chile, Costa Rica, Brazil, and Honduras before the regional elimination initiative was adopted by the directing council of PAHO. These experiences showed that implementing strategies and recommendations to eliminate rubella and congenital rubella syndrome was feasible. In addition, rubella elimination provided opportunities for strengthening the health system by building partnerships and involving national and local authorities (Table)

**Table:** Rubella and congenital rubella elimination^a,b^

Time	Country	Target group	Vaccine used	Coverage achieved (%)
1998–2001	English-speaking Caribbean 18 countries	2.16 million men/women 20–29 y	MR and MMR	Average 80^c^ Range 64–97
1999	Chile	2.5 million women 10–29 y	R	98
2001	Costa Rica	1.6 million men/women 15–39 y	MR	98^c^
2001 2002	Brazil 13 states 11 states	27 million, women 12–39 y^d^	MR	95
2002	Honduras	3.3 million men 5–39 y women 5–49 y	MR	98 Men 95 Women 98
2004	El Salvador	2.8 million men/women 15–39 y	MR	99 Men 93 Women 99
2004	Ecuador	4.8 million men/women 16–39 y	MR	100 Men 100 Women 100

^a^CRS, congenital rubella syndrome; R, rubella containing vaccine; MR, double viral vaccine, measles/rubella containing vaccine; MMR, triple viral vaccine. ^b^Source: *12**–**18*. ^c^Men/women vaccinated in equal proportions. ^d^Some states modified the age of the group based on the year of the vaccine introduction.

In the English-speaking Caribbean, the cost-benefit ratio for vaccination was 13.3:1, and the cost-effectiveness was U.S.$2,900 per prevented case of congenital rubella syndrome. The comprehensive vaccination plan for adults included strategies for safe injections and monitoring adverse events during campaigns. In Chile, health promotion was well-planned, and awareness among women about their own health and their family's increased.

In Costa Rica, the decision to conduct the campaign was based on epidemiologic information and cost benefit analyses. The president of the republic declared the event official by executive decree, which greatly facilitated intersectoral coordination of public and private institutions. The strategies to reach older populations in urban and rural areas differed. In urban areas, vaccination teams began with captive populations and ended with door-to-door "mop-ups." In rural areas, the campaign began in more isolated places and moved towards city centers with door-to-door "mop-ups" or micro-concentration. Enlisting the participation of medical societies and professional associations and the active involvement of health workers were essential. Blood banks had to be involved so that the nation's blood supply was not threatened. The monitoring of post-vaccination adverse events and immediate investigation of events was considered a top priority. Extensive follow-up studies of women who were vaccinated and not known to be pregnant demonstrated that an increased risk for adverse events did not exist.

In Brazil, social mobilization was the key to the success of a vaccination campaign aimed at adults. Health professionals recognized as leaders and decision makers in the country were called upon to explain the campaign and clarify issues. A rapid response plan was put together in each state to deal with crises or adverse events of vaccination, and a telephone hotline was set up for the public. The most common questions were where to go to be vaccinated (36%), what to do if a pregnant woman had been vaccinated by mistake (14%), and other questions about adverse events (10%). Health authorities used the rapid evaluation tools to determine which groups of women had not been vaccinated and designed effective methods to reach them. In Honduras, adult men in marginal areas rarely seek health services, so the campaign served to increase health contacts.

In El Salvador, vaccination methods implemented during the first 2 weeks were aimed at captive populations in work and study centers and in high-transit areas or areas with high populations. House-to-house vaccination was continued after normal work hours to ensure that the adult population was at home. In addition to promotion during the campaign, the plan for social communication and mobilization launched activities aimed at motivating blood donors and avoiding blood shortages before the campaign.

In Ecuador, a presidential decree urging public and private sectors to participate was helpful. A total of 6,722 rapid coverage monitoring surveys were performed in 2,006 health units, and 75% of municipalities reported coverage >95%. When coverage was not recorded, the surveys helped to identify susceptible groups and implement "mop-up" activities. The social communication showed that the source of information from health units, TV, and radio was most effective. School source information was also helpful.

## Surveillance

To accelerate the strategies to eliminate rubella and congenital rubella syndrome, countries were challenged to improve surveillance of congenital rubella syndrome, while strengthening the already established integrated measles and rubella surveillance system. Improving congenital rubella syndrome surveillance required that health authorities more effectively identify and monitor women of childbearing age who may have contracted rubella during their pregnancy.

The reporting network of Latin America and the Caribbean includes nearly 22,000 reporting sites. The network encourages collaboration between the public and private sectors. The network has contributed to the formation of trained epidemiologists with experience in surveillance, disease control, and operations research to respond to new, emerging, and reemerging infectious diseases, such as cholera, influenza, and yellow fever (*19*). The surveillance work has also contributed to women having more frequent contact with prenatal services.

Implementing congenital rubella syndrome surveillance is particularly important during the initial phase of elimination to monitor effectiveness of congenital rubella syndrome prevention. Moreover, all countries are setting up consultation teams in hospitals for congenital infections with the active participation of neonatologists. Well-baby check-ups are used to look for congenital malformations. As a by-product, national registries for identifying all congenital malformations and ensuring their follow-up are being strengthened.

## Discussion

Strengthening health services by implementing high-quality congenital rubella syndrome surveillance, periodic well-baby check-ups, or setting up areas for consultations for congenital infections is essential for high-quality, comprehensive perinatal care. The use of these perinatal information systems improves the monitoring of children with congenital malformations and provides them with increased contact with rehabilitation and special education services. The by-product of such efforts is providing patients with better, more specialized care and referrals.

Empowering women with the knowledge of prevention is a key strategy for improving the quality of health care for women (*20*). The communication and social mobilization strategies of the rubella and congenital rubella syndrome elimination initiative are designed to enable women to make their own choices, exercise their rights, and demand improved health care.

Regional perinatal information systems, such as the Latin-American Center for Perinatology and Human Development and the Congenital Malformation Latin-American Collaborative Study, help to improve surveillance and services for newborns (*21*). These systems also provide valuable information on maternal deaths and are important in investigating all maternal deaths.

The rubella elimination initiative offers an opportunity to put adults in more frequent contact with the health services. In some communities, adult men also make key decisions affecting care-seeking for women and their newborns. Thus, men must be aware of women's needs, risks, and warning signs. Promoting men's roles as partners and fathers is essential for enlisting their participation and support, a message that is always conveyed during adult vaccination campaigns (*22*). In some communities, mothers may more easily gain access to specific health services if they have the full support of other family members, especially their husband.

A major challenge in women's and perinatal health care is ensuring universal access, which means bring healthcare services high-risk communities where poor and underserved groups live. Because the vaccination campaign is aimed at 100% of the population, inequities based on sex, ethnicity, social class, race, and geographic distribution are reduced. This experience also greatly contributes to the reduction of inequities of maternal health outcomes (*23*).

To improve the health of all persons, scientists, health providers, administrators, business, media, and religious leaders, and all other sectors of society must be enlisted. The most fundamental challenges faced by the rubella elimination initiative are the mobilization and coordination of all sectors in a joint cause. Promoting women's health care and safe motherhood should be well grounded in a multisector approach that requires carefully building and maintaining partnerships.

Immunization services aimed at protecting children have played a decisive role in improving childhood health in the Americas. The foundation for expanding vaccination to other age groups should generate opportunities for strengthening health services for adults. Countries in the Americas are working hard to extend the benefits in the fields of social mobilization, community participation, staff education, epidemiologic surveillance, and program management. Countries are also committed to ensuring that the rubella elimination initiative plays a major role in improving women's health care.
